# Factors That Affect the Adoption of Fertilizer Among Cocoa Farmers in Developing Countries: A Review

**DOI:** 10.1002/gch2.202400175

**Published:** 2024-12-07

**Authors:** Rebecca Alice Wokibula, Katherine Dentzman, Francis Akitwine, Johnson Godlove Mtama, Moureen Mbeiza, C. Lee Burras

**Affiliations:** ^1^ Soil Science‐Department of Agronomy Iowa State University 716 Farm House Ln, 1025 Ames IA 50011 USA; ^2^ Rural Sociology and Rural Public Policy Department of Sociology and Criminal Justice Iowa State University 510 Farm House Ln, 303D Ames IA 50011 USA; ^3^ Iowa State University ‐ Uganda Program P.O. BOX 218, Kamuli Uganda

**Keywords:** cacao, characteristics of innovations, cocoa production, diffusion of innovations, farmer decision‐making, fertilizer adoption, sub‐saharan Africa

## Abstract

Sub‐Saharan Africa's adoption of inorganic fertilizer lags behind other developing nations, creating limitations for small‐holder cocoa producers. Using the Diffusion of Innovations (DOI) Theory, articles assessing inorganic fertilizer (non)adoption by cocoa producers in Sub‐Saharan Africa are reviewed. Factors influencing adoption fell into two major categories: socioeconomic characteristics of the potential adopter and characteristics of the innovation itself. Farm income, access to credit, labor requirements, and fertilizer price/availability are especially influential. Future studies and outreach efforts to consider the interplay of many personal, agronomic, and technological factors when promoting fertilizer adoption in cocoa farming are encouraged.

## Graphical abstract

Sub‐Saharan Africa's adoption of inorganic fertilizer lags behind other developing nations, creating limitations for small‐holder cocoa producers. Using the Diffusion of Innovations (DOI) Theory, articles assessing inorganic fertilizer (non)adoption by cocoa producers in Sub‐Saharan Africa are reviewed. Factors influencing adoption are organized into two major categories: socio‐economic characteristics of the potential adopter and characteristics of the innovation itself.

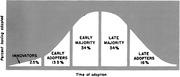

## 1 Introduction

Declining soil fertility and soil productivity induced by nutrient mining through continuous cultivation of small‐scale, low‐input (fertilizer) farming activities is a recurrent concern in most developing countries in sub‐Saharan Africa.^[^ ^1^, ^2^, ^3^ ^]^ One strategy to stop this ongoing soil degradation is encouraging farmers to embrace new technology, particularly inorganic fertilizers, that boost crop yields. In places where cocoa is produced, it has been observed that using inorganic fertilizers considerably increases cocoa productivity.^[^ ^4^, ^5^ ^]^ Ref. [6] on‐farm experiment, cocoa yields reached 1890 kg ha^−1^ after 2 years of fertilizer treatment in Ghana and Cote d'Ivoire compared to 765 kg ha^−1^ without fertilizer. Another study in Cote d'Ivoire by Alexis et al.^[^ ^7^ ^]^ discovered that cocoa yield increased from 600 to 1000 kg ha^−1^ in the third year after fertilizer treatment.

However, Sub‐Saharan Africa's inorganic fertilizer use still lags behind that of other developing nations, where the intensification of production agriculture has been followed by a marked rise in fertilizer application.^[^ ^8^ ^]^ Aneani et al.^[^ ^9^ ^]^ reported a fertilizer adoption rate of only 33% for cocoa farmers in Ghana, while Ruff and Bini^[^ ^6^ ^]^ also noted that although roughly 70% of Ghanaian cocoa producers buy fertilizer, the intensity of fertilizer application is low with an approximate estimate of 90 000 tonnes versus the potential requirement of ≈350 000 tonnes. According to Okoboi & Barungi,^[^ ^2^ ^]^ the adoption rate of inorganic fertilizer among small‐scale farmers is as low as 8% for various crops, at application rates as low as 1 kg of nutrient per hectare per year.^[^ ^10^ ^]^

This low rate of adoption of fertilizers by farmers in sub‐Saharan Africa has inspired a field of literature on the factors that affect fertilizer adoption among farmers in developing countries. However, most research published in agricultural journals constitutes socio‐economic factors as characteristics pertaining to the individual, leaving out factors pertaining to the characteristics of the innovation and factors that enable a linkage between the individual and the innovation. Furthermore, understanding how an innovation's adoption progresses over time is a valuable concept (the Diffusion of Innovation (DOI) theory) but is still rarely addressed in agricultural‐based literature. This research fills this gap by highlighting the interconnectedness of some aspects of the DOI theory and an array of factors (individual‐based and innovation‐based) that affect adopting an innovation (fertilizer).

Knowing the DOI theory provides a deeper understanding of the fertilizer adoption trends (length of adoption cycle), the linkage between stages of adoption and characteristics of adopters, paves the way for reevaluation of non‐adoption, and looking at the interconnectedness of factors that hinder adoption may pave the way for the promotion of specific enabling environment (considering target audiences for an innovation, conducting prior and post‐training) to enhance adoption. Considering the interconnectedness of factors and how adoption progresses may lead to more realistic and holistic recommendations and interventions for achieving increased adoption and designing and implementing better fertilizer use‐related policies for the cacao production sector.

This literature review aims to highlight the DOI theory and compile and discuss 13 factors using cocoa farming as a case study. These factors will be organized into two categories: 1) the socio‐economic characteristics of adopters, in this case, the cocoa farmers, and 2) the characteristics of an innovation, in this case, the fertilizer. We follow the literature review by identifying overarching trends, conclusions, and recommendations for improving future studies.

## 2 Diffusion of Innovation (DOI) Theory

To better understand the literature on fertilizer adoption rates in developing countries, it is helpful to understand and draw insights from a commonly used framework concerning adopting new technologies generally—DOI theory. A social science theory developed by E.M. Rogers in 1962, DOI explains how an idea seen as novel reaches individuals in a community through set communication pathways.^[^ ^11^ ^]^ It involves understanding when the innovation was introduced, who introduced it when it was adopted, and what the consequences of adoption are. This process collects, integrates, and evaluates information that can help prospective adopters make informed decisions. This diffusion process results in people adopting a new idea, behavior, or product as part of a social system.

Nonetheless, the adoption of an innovation by the potential users does not simultaneously happen all at once but occurs in phases depending on where the farmer falls on a scale ranging from early adopter to laggard, irrespective of the innovation's superior traits;^[^ ^12^, ^13^ ^]^ this is due to uncertainty about the functionality, unanticipated dangers, and technicalities of the new idea (the process of diffusion as a disequilibrium process),^[^ ^14^ ^]^ as well as variation of the adoption benefits over the potential adopters due to their behavioral and structural differences (see, e.g., ref. [15]).

**Figure 1:**
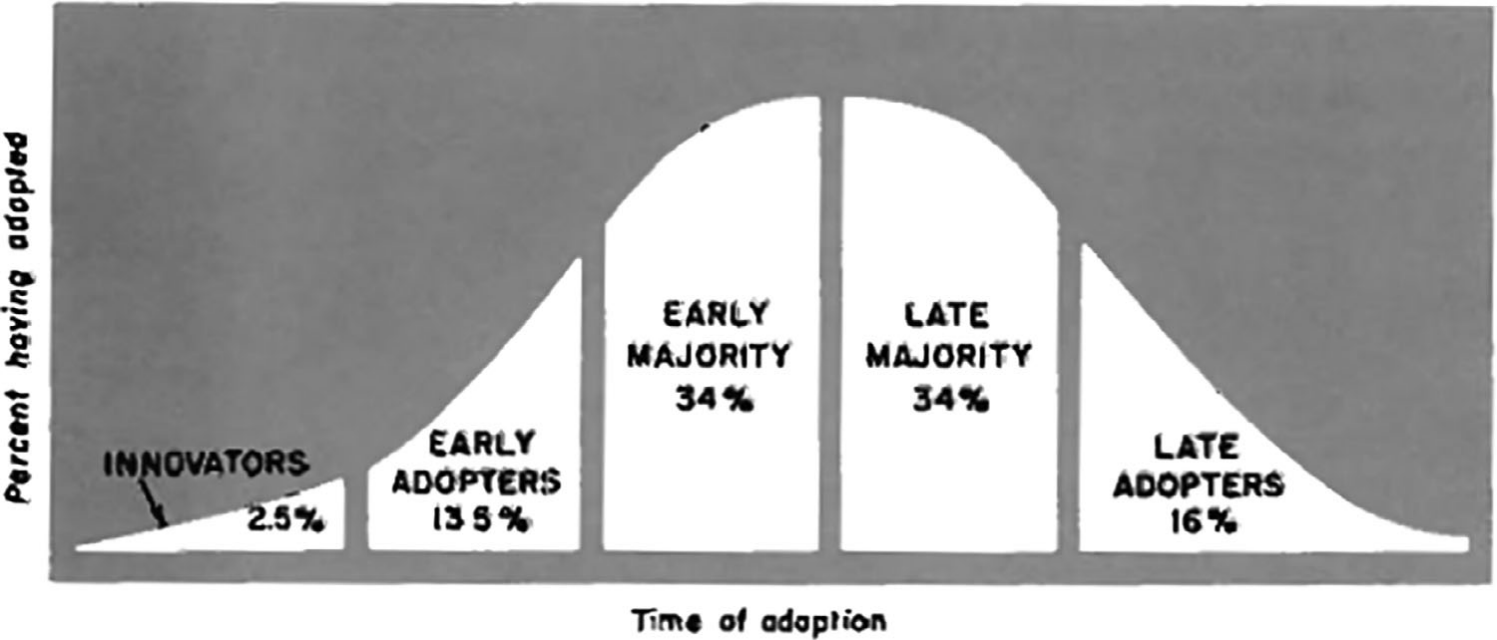
Distribution of farmers among the five categories according to time of adoption.^[^ ^12^ ^]^ This work is licensed under a Creative Commons Attribution‐NonCommercial‐NoDerivs 3.0 License.

The DOI theory outlines five different categories of adopters,^[^ ^16^ ^]^ as represented in **Figure** **1**.^[^ ^12^ ^]^ It shows how adoption decisions are made in waves and over a period of time, with innovators being the first to try new ideas and technologies due to being invested in new concepts, adventurous and risk‐taking, motivated by the idea of being change agents, financially well off, and operating in more cosmopolitan social circles.^[^ ^12^, ^16^ ^]^ Early adopters provide opinion leadership, want to be first, and desire to be at the forefront as examples to others. The opinion leaders take advantage of the situation to effect change, tend to be integrated into the local social system, have social status, and can exercise concentrated influence in their area.^[^ ^12^, ^16^ ^]^

The early majority, who adopt a given technology directly following innovators, are very social, have deliberate contact with peers, and are willing to change their behavior if it enhances their quality of life and productivity.^[^ ^12^, ^16^ ^]^ They want proven ideas and technology, so they need new ideas and tech vetted by peers and colleagues. The late majority, in contrast, are skeptical and tend to adopt ideas later than the average person in a given social system. When they do adopt an innovation, it is often out of social or economic necessity due to pressure from system norms.^[^ ^16^ ^]^ Laggards tend to be older, are focused on traditions, often have limited socializing, are more‐or‐less only in contact with family and close friends, and are thus more relaxed to adapt. They view innovators and innovations with suspicion, so they adopt them very late, so the original innovators may well consider the innovations obsolete.^[^ ^12^, ^16^ ^]^

This broad categorization represents the early stages of DOI theory; more recently, it has been expanded to emphasize a range of socio‐economic characteristics of potential adopters and characteristics of the innovations themselves, which in this review form the main subject.

## 3 Methods

The methodology involves synthesis, evaluation, identification of relevant studies, and compilation of the available literature on the factors that affect fertilizer adoption among cocoa farmers in developing countries. A thorough investigation was carried out using internet databases of Google Scholar from inception to 2022. The following keywords and phrases were used in the search: “cacao production,” “fertilizer adoption,” “inorganic fertilizer use in developing countries,” “adoption of innovation,” “characteristics of innovations,” “social, economic characteristics of adopters,” “technology transfer,” “DOI theory,” “factors affecting adoption of innovations,” “determinants of fertilizer adoption,” “cacao production systems,” and “factors influencing adoption decisions” In addition, a manual search was carried out by going through the reference lists of relevant articles. Sections from the reference materials and Google searches that addressed the purpose of the review in their titles and abstracts were compiled and used for the review. All information that addressed the purpose of the review should have been included. A total of 69 studies were identified and used.

The 13 factors were selected to provide a scope to the study and were selected as representative factors that affect the adoption of an innovation (fertilizer) from the literature of papers considered in this review. The 13 factors were selected to include the two categories of the socio‐economic characteristics of individuals and innovation (fertilizer) characteristics.

In the literature search, the word categories used generated so many papers of which approximately more than 100 papers were perused. However, the 69 papers included in the review were deemed by the author as sufficient in addressing the subjects of the location of cacao farms (Africa), the technology discussed (Fertilizer use), the thirteen factors selected, and the DOI theory explained. All 69 papers align with the adoption of fertilizer and other related innovations used by farmers in related contexts.

## 4 Socio‐Economic Characteristics Impacting Adoption

Early studies on the Diffusion of Innovations focused on characteristics of individual farmers and their farms, citing how potential adopters clustered along the adoption timeline due to common traits, including attitudes, values, abilities, group memberships, and social status.^[^ ^12^ ^]^ This line of research has developed over time to include six primary groups of socio‐economic farmer characteristics influencing adoption: 1) profit expectations and income, 2) off‐farm income access or reliance, 3) property and farm size, 4) age, 5) education, and 6) values, norms, and beliefs.^[^ ^17^ ^]^ Of course, other traits also matter—studies have identified social networks, aesthetics, gender, political views, risk preferences, and many more socio‐economic factors as having a significant impact on adoption behavior in agriculture.^[^ ^18^, ^19^, ^20^, ^21^ ^]^

Below, we expand on several socio‐economic farmer and farm characteristics research has identified as particularly relevant to fertilizer adoption among cocoa growers in developing nations. We group this discussion into the following categories: perceptions, access to information, farm size, farmer age, farm income, access to credit, and farmer gender.

## 5 Perceptions (Values, Norms, and Beliefs)

The Britannica dictionary defines perception as how one thinks about or understands something. Perceptions are synonymous with values, attitudes, norms, beliefs, and opinions, among others. Perceptions may be inherent in an individual or may be externally constructed depending on the society, cultural, economic, and biophysical spatial factors, among others.^[^ ^22^ ^]^ Perceptions are thus worth considering when answering questions on factors that influence people's actions like in the event of adoption of innovations. For instance, farmers' choices of practices are influenced mainly by their way of thinking ‐perceptions.^[^ ^22^ ^]^

The degree to which farmers will adopt new technology depends on how they perceive it in the context of their limitations. For instance, innovations perceived as riskier or more uncertain have lower adoption chances, whereas those seen as effectively addressing a restriction have greater adoption odds.^[^ ^23^, ^24^, ^25^ ^]^ This perception of risk is influenced by an individual's characteristics, such as being a relatively resource‐poor small‐holder.^[^ ^20^ ^]^ Furthermore, prevailing economic, biophysical, and societal conditions may limit farmers' abilities and capacities to adopt innovations, warranting the benefits of aligning present capabilities with innovations.^[^ ^22^ ^]^

Social norms can also function to increase or depress adoption rates; the influence of locally respected farmers, family, friends, and neighbors can significantly influence a person's choice to choose a specific technology, with late adopters in particular influenced by the perceptions and pressures from people in their social network.^[^ ^20^ ^]^ Values such as environmental sustainability have also been shown to impact adoption, as have *beliefs* about the effectiveness of a given practice (as opposed to the objective effectiveness).^[^ ^20^ ^]^ Although this area of research has been minimally studied, there is still some evidence that perceptions, including farmers' values, aspirations, norms, and beliefs (as influenced by their social position and network), can have a significant impact on adoption decisions.^[^ ^17^, ^20^, ^22^ ^]^

Perceptions can be particularly relevant when an innovation reflects or flouts widespread cultural and societal values or norms—for example, it has been found that Amish communities adopted sustainable agricultural practices faster than non‐Amish communities due to cultural values aligned with land and soil protection.^[^ ^26^, ^27^ ^]^ Alignment of practice with cultural values confers social status on adopters, further encouraging and speeding adoption.^[^ ^26^ ^]^ Homogeneity of social groups can also hasten diffusion, as can broader cultural values such as risk acceptance, cosmopolitanism, authoritarianism, individualism, and competitiveness.^[^ ^26^, ^27^ ^]^ These cultural values, related beliefs, and norms significantly influence potential adopters' perceptions of the innovation and potential adoption.^[^ ^27^ ^]^

One prevailing positive perception of fertilizers among farmers in underdeveloped nations is that they are a miracle solution that boosts crop yield.^[^ ^4^ ^]^ However, this is undermined by additional beliefs that fertilizers are costly, do not provide a return on investment, and, if applied excessively, damage the soil and the environment.^[^ ^28^ ^]^ Perceptions about soil fertility will also influence the decision to adopt fertilizer. While some cocoa farmers said they do not use fertilizer because they think the cocoa trees' and their leaves' decomposition on the soil will increase its fertility,^[^ ^29^ ^]^ there are others who believe that the soils are so old and can no longer have any productivity for sustainable crop production.^[^ ^23^, ^24^ ^]^ Ref. [22] emphasize that knowledge and positive beliefs about a given technology may demystify the negative perceptions toward its adoption.

The nonuse of fertilizer in traditional cocoa production systems, which were majorly agroforestry, may also shape farmers' perceptions to date. An example is Cameroon, which has managed to keep cocoa plantations for many years without fertilizer use but by replacing old and dying trees with young ones.^[^ ^30^ ^]^ Cocoa and shade trees grown together in perennial systems without fertilizer continued to give good yield without excessive soil degradation,^[^ ^31^ ^]^ or noticing a decline in cacao yield.^[^ ^32^ ^]^ These diverse agroforestry systems foster enhanced biological processes for nutrient recycling and efficiency for improved soil fertility and heterogeneity of cocoa stands.^[^ ^30^ ^]^ It is with these previous trends in perennial agroforestry systems that farmers who do not use fertilizer today base, and continue to think the cocoa trees' and their leaves' decomposition on the soil will increase its fertility,^[^ ^29^ ^]^ even amid declining soil fertility under nonagroforest cocoa systems.

Perceptions and other factors that determine the adoption of an innovation change with time and information, as does the adoption status. Pannell et al.^[^ ^17^ ^]^ states that uncertainty about innovation is high in the early phases of the diffusion and adoption process, while the quality of decision‐making may start low but reduce as time allows for more informed decisions. Understanding how perception over time affects technology adoption decisions makes it possible to provide information and implementation strategies that are specifically tailored to the demands, settings, and socio‐economic circumstances of producers at various phases in the adoption process.^[^ ^33^, ^34^, ^35^ ^]^ Such strategies may include, for instance, educating farmers on the proper rates, quantities, and conditions (soil, crop, and climate) for fertilizer use to produce the best yields, connecting them with the appropriate contacts (information, partners, businesses), and creating an enabling environment in terms of policy and regulations.

## 6 Access to Information/Extension

Information availability and accessibility are crucial factors in determining whether a technique will be accepted.^[^ ^36^, ^37^ ^]^ As knowledge is created during the innovation implementation process and gradually circulates among the potential adopters, the number of adopters of the innovation rises.^[^ ^13^, ^14^ ^]^ Information increases familiarity with a new idea and can influence perceptions of risk, facilitating adaptive behavior^[^ ^26^ ^]^; it is, therefore, no surprise that more difficult adoption decisions result in potential adopters seeking more information from a wider variety of sources.^[^ ^17^ ^]^

The source of information a farmer uses is also essential; several studies have found that interpersonal contact between farmers, conservation agencies, fertilizer dealers, extension educators, and certified crop advisors is key for influencing the adoption of new practices.^[^ ^20^, ^33^ ^]^ While media plays a vital role in initial awareness, information from close peers in social and organizational networks is the most influential in adoption decisions.^[^ ^12^, ^17^, ^26^, ^38^ ^]^ Therefore, an individual's position within interpersonal and organizational social networks, along with social density and physical proximity, is key in determining adoption.^[^ ^17^, ^33^ ^]^ Further, confidence and adoption likelihood greatly increase when interpersonal, media, and scientific information sources provide repeated and consistent messaging.^[^ ^17^ ^]^ Generally, individuals with easy access to sources of quality, detailed information on new agricultural technologies or practices—and who trust these sources—tend to adopt a given innovation more quickly.^[^ ^20^ ^]^

This is in line with Nayenga^[^ ^39^ ^]^ who claimed that having access to extension information enhances the possibility that people will use fertilizer. Information from a qualified expert or reliable source strengthens a farmer's decision‐making ability since it eliminates uncertainty and is a crucial strategy for changing unfavorable opinions.^[^ ^4^ ^]^ These findings agree with^[^ ^13^ ^]^ about innovators making more use of external sources of information other than waiting for extension agents. Anang^[^ ^4^ ^]^ showed that adopters had less contact with extension agents, which is consistent with the characteristics of the innovators and early adopters concerning their position on a spectrum from innovators to laggards who reported that innovators want to be the first to try the innovation, are venturesome, interested in new ideas and very little, if anything, needs to be done to appeal to this population.^[^ ^13^, ^25^, ^40^ ^]^

## 7 Farm Size

Generally, studies have found that producers operating larger farms tend toward higher adoption of recommended technologies and best management practices.^[^ ^12^, ^17^, ^20^ ^]^ The driving reason behind this trend has multiple possible explanations, from higher education and social connectivity to more readily available investment capital.^[^ ^12^, ^20^ ^]^ Larger farms may also have increased capacity and greater potential benefits, given their economy of scale.^[^ ^12^, ^17^, ^33^ ^]^ Smaller farms, in contrast, struggle to adopt new technology due to a scarcity of resources or perceived minimal returns to small‐scale operations. Small farms may also be operated by farmers with less knowledge or experience; however, there is evidence that small farms are more driven by environmental and noneconomic goals, complicating the relationship between farm size and the adoption of various agricultural technologies and practices.^[^ ^20^ ^]^

Despite some disagreement on the direction of the impact, farm size is often a determinant factor of the adoption of agricultural technologies, including fertilizer,^[^ ^9^, ^29^ ^]^ and farm size coupled with high prices of fertilizer has been identified as a disincentive to adoption. Regarding the farmland allocated to cocoa production, the mean farm size of the adopters is significantly lower than the nonadopters.^[^ ^5^ ^]^ However, in the Ghanaian rural economy, farmland ownership is considered a valuable asset, as it may favor farmers with big cocoa plantations regarding fertilizer uptake and intensification. Similarly, the value of productive farm assets had a strong and direct relationship with fertilizer adoption probability but not intensity, with larger enterprises adopting technology sooner.^[^ ^15^ ^]^ This supports the diffusion model's assertion that large (but not excessively large) farms are the most inventive (e.g., ref. [15]). This may be due to larger farms having more access to risk‐bearing financial resources to invest in innovation; they can easily make profits from innovation due to increasing returns to scale and might benefit more through division of labor and labor specialization. For instance, the size of the cocoa farm is anticipated to have a favorable impact on adoption because as the farmer devotes more of his total accessible land to cocoa cultivation, the cocoa yield and income would likely increase, increasing the likelihood of technology adoption.^[^ ^9^ ^]^

The results above diverge from^[^ ^4^ ^]^ who found a negative link between farm size and fertilizer adoption for cocoa producers in Ghana. Farmers who produce cocoa on small farms in Ghana and Cote d'Ivoire are more likely to use fertilizer than those who do so on larger farms because an increase in farm size reduces the likelihood of doing so.^[^ ^41^ ^]^ This could be because purchasing fertilizer for larger farms requires more investments than for small ones.^[^ ^29^ ^]^ Another possibility is that farmers with huge farms can still harvest their large plots of land if they continue to use fundamental agronomic techniques rather than fertilizer.

## 8 Age of Farmer

While age does appear to have an impact on rates of adoption, there is mixed evidence, with some studies finding that younger farmers adopt best management practices at a higher rate than older farmers,^[^ ^12^ ^]^ while others find no difference.^[^ ^20^, ^33^ ^]^ Prokopy et al. hypothesize that age will have a negative impact on adoption, citing research that has shown older farmers are less likely to adopt grazing practices, soil management, manure testing, and conservation investments. Indeed, their analysis of 46 studies including age as a variable predicting adoption found that age was more frequently a negative than positive predictor—although this was not significantly different from what could be observed by chance.^[^ ^17^ ^]^ Speculate that older farmers have less incentive to invest in practices that may not pay off in their lifetimes and could also be limited in their adoption choices by declining physical health. It is additionally possible that positive associations of adoption with age could be due to the correlation of age with years of experience in farming.^[^ ^33^ ^]^ Overall, the literature is highly mixed with inconsistent findings.^[^ ^17^, ^20^ ^]^

According to ref. [4] age substantially impacted farmers' decisions to use fertilizer in cocoa cultivation in Ghana, with younger farmers more likely to accept technological change. However, refs. [41,42] reported a positive significant influence of age on fertilizer adoption by cocoa farmers in Ghana and Cote d'Ivoire, respectively, which is consistent with.^[^ ^43^ ^]^ The negative correlation would result from age being a proxy for farming experience: experienced farmers are less likely to adopt fertilizer compared to relatively inexperienced farmers as they have more alternative knowledge to rely on to make the business profitable than adopt an innovation.^[^ ^44^ ^]^ Furthermore, older farmers in developing countries are more likely to have larger households, hence more financial obligations, adversely affecting their adoption decisions^[^ ^42^ ^]^ estimated that a cocoa farmer's age will negatively affect adoption because as he gets older, his physical strength tends to decline, which is thought to have a detrimental effect on the adoption of the technology.

However, the positive correlation between age and fertilizer adoption would still rely on the vital role of farming experience in adopting agricultural technology practices.^[^ ^45^ ^]^ Experience provides more insights about both the crop and technology interaction and provides a certain way for decision‐making. With long experience comes repeated failures in the past of experienced farmers, hence a motivation to try modern, improved technologies. Farmers with more experience in cocoa cultivation could apply their cropping experience in cocoa cultivation, which could increase their ability to adopt cocoa technologies. This echoes broader findings that positive and negative relationships exist between age and agricultural technology adoption.^[^ ^25^ ^]^

## 9 Farm Income

Income, like farm size, correlates positively with farmers' adoption rates. Those with high levels of income and capital may be less risk‐averse, more willing to invest in new technologies or practices, and relatively insulated from the consequences of uncertain outcomes.^[^ ^20^ ^]^ Further, costs, including capital and opportunity costs, which pose significant barriers to adoption, are substantially reduced by high farm income levels. Income is, therefore, more often seen as a barrier to adoption (when low) rather than an incentive to adopt (when high).^[^ ^17^, ^33^ ^]^ In their review ref. [33] found that most studies indicated a positive and significant association of farm income with adoption, positing that higher income is associated with greater capacity. Access to off‐farm income can also have an impact, increasing financial security that would allow for experimentation with adoption while also, conversely, decreasing adoption of practices involving greater management demands.^[^ ^17^ ^]^

Fertilizer adoption is positively impacted by the value of productive farm assets and off‐farm income.^[^ ^43^ ^]^ High revenues offer farmers the ability to purchase fertilizer, increasing its uptake. The adoption of fertilizer by cocoa farmers in Ghana was positively and extremely substantially correlated with farm revenue, with adopters experiencing significantly greater farm income.^[^ ^4^ ^]^ This is in line with,^[^ ^46^, ^47^ ^]^ which found that men are more likely to use fertilizer than women in Uganda since they earn, on average, more money.

## 10 Access to Credit

The price or cost of fertilizer has been mentioned throughout the literature as one of the reasons farmers are not using fertilizer.^[^ ^2^, ^22^, ^29^, ^48^ ^]^ The situation is further worsened by the lack of credit facilities toward fertilizer access.^[^ ^20^, ^22^, ^48^, ^49^, ^50^, ^51^, ^52^ ^,]^

Availability of timely credit is a key determinant or factor of the adoption of agricultural technologies. For instance, lack of access to credit while waiting for government reimbursements has been identified as a key barrier to adopting conservation programs in the U.S.^[^ ^20^ ^]^ Small‐holder farmers in developing countries are similarly more risk‐averse when their access to credit is limited^[^ ^20^, ^33^ ^]^ review finds that monetary measures of access to capital, including credit, positively correlated with adoption.

Awareness of various sources of credit has also been cited as impacting adoption decisions; in rural areas, local moneylenders may complicate this by charging exorbitant interest rates.^[^ ^49^ ^]^ Access (via the Internet) to credit schemes offered by large professional banks could be key in allowing farmers to access reasonable credit rates and increase adoption.^[^ ^49^ ^]^ Access to cooperatives may also be key in providing credit, especially to small‐holder farmers.^[^ ^53^ ^]^ For instance, ref. [51] found that, in Indonesia, the number of credit sources available to farmers and the ease or simplicity of accessing credit were among the strongest predictors of chili farming adoption.

Credit availability was also listed by Green and Ng'ong'ola^[^ ^50^ ^]^ as a primary influence on Malawians' adherence to fertilizer recommendations. Access to credit in cash is predicted to positively influence fertilizer adoption because it increases the capability to purchase the fertilizer. Morris et al.^[^ ^52^ ^]^ claims that even if farmers think fertilizer is profitable, they may be unable to buy it if they lack funds or credit. These findings are supported by the reasoning that most farmers in developing countries need access to credit to buy fertilizer and market their production^[^ ^48^ ^]^ due to a lack of adequate collateral matched to the amount of credit. However, fertilizer adoption may still not increase even with increased access to credit due to competition with other farm activities, such as pests and disease control, for the limited credit.^[^ ^54^ ^]^

## 11 Gender of a Farmer

Farmers’ gender has been found to have mixed effects on adoption rates.^[^ ^33^ ^]^ In developing countries, for example, adoption rates by gender vary depending on women's control over farm decision‐making.^[^ ^20^ ^]^ Some studies have found that women have more positive attitudes toward collaboration and certain types of agricultural technologies, especially conservation‐related ones, than men—yet they also have lower levels of knowledge about these technologies and how to implement them.^[^ ^20^ ^]^ Gender may also have a moderating role in other factors influencing adoption. For example, some studies have found that women farmers are more likely to learn about—and subsequently adopt—a new technique when the messenger promoting it is also a woman,^[^ ^20^ ^]^ demonstrating the impact of gender on how information sources encourage or discourage adoption. Men and women also tend to have different communication patterns and learn about agricultural innovations in different ways^[^ ^55^, ^56^ ^]^; in general, gender appears to often be a moderator impacting knowledge acquisition, relative advantage, ease of use, compatibility, and other direct predictors of adoption.^[^ ^20^, ^57^ ^]^

Because most of the tasks involved in producing cocoa demand physical effort and are labor‐intensive, men are more likely to work in this industry than women.^[^ ^29^ ^]^ Research by ref. [58] on cocoa producers in Ghana's Eastern Region revealed that 78% of cocoa farmers were men and 22% were women. This is also consistent with,^[^ ^59^ ^]^ with (37.4%) adoptions for males versus (28.3%) for females. Men farmers are more likely to adopt fertilizer than their women counterparts.^[^ ^43^ ^]^ For Uganda, and maybe other developing countries, men's household heads have a higher income than women.^[^ ^46^, ^47^ ^]^ This could result in men having more access to valuable resources such as land and credit than women, hence in a better position to adopt new technologies.^[^ ^39^, ^46^, ^47^, ^60^ ^]^ The increased access to extension services^[^ ^39^ ^]^ could result from the male‐dominated sector.^[^ ^61^ ^]^ Other reasons could be due to higher academic attainment and years of experience. With some enterprises like cocoa production being more male‐dominated, more male farmers are expected to adopt technologies than their female counterparts, other things being equal.

## 12 Overview of Characteristics of the Innovation

As mentioned, the adoption of an innovation by the potential users does not happen all at once among adopters but occurs in phases depending on whether the farmer is an early adopter or a laggard^[^ ^13^ ^]^ due to uncertainty about the operating conditions, risks, and performance characteristics of the new technology (the process of diffusion as a disequilibrium process).^[^ ^14^ ^]^ The characteristics of innovation can impact the process of adoption, and according to refs. [23, 62] and [25, 40] there are five characteristics of innovation to help explain factors that impact the rates of adoption: relative advantage, trialability, compatibility, complexity, and observability.^[^ ^12^ ^]^ Relative advantage and trialability have been known to encompass the three; hence can be seen as having a great influence on the chances of adoption.^[^ ^17^ ^]^ According to Rogers^[^ ^25^ ^]^ more people will be interested in adopting a technique with both a high relative advantage and high trialability.

The term “relative advantage” refers to “the extent to which an invention is viewed as being superior to the idea [or practice] it supersedes,” the perceived net advantages if one adopts it, and is dependent on the specific goals of the landowner and the biophysical, economic, and social environment in which the innovation will be applied; such as the innovation's short‐term input costs, yields, and output prices, or other activities that it affects.^[^ ^25^ ^]^ It is the decisive factor determining the ultimate level of adoption of most innovations in the long run. Relative advantage further depends on a range of economic, social, and environmental factors, such as the short‐term input costs, yields, and output prices of the innovation.^[^ ^63^, ^64^ ^]^

Trialability is the characteristics of the innovation impacting the ease with which the landholder can learn about its performance and optimal management‐ or how easy it is to shift from nonadoption to adoption via a learning cycle. Trialability goes beyond the ease of physically establishing a trial to include factors that influence the ability to learn from a trial, such as the complexity of the issue being addressed.^[^ ^12^, ^17^ ^]^

Trialing an invention gives data that lessens uncertainty regarding the relative benefit of the method^[^ ^65^ ^]^; it can increase the probability of the landholder making a correct decision and provides an opportunity for the landholder to learn the skills needed to apply the innovation. Its small‐scale nature makes it possible for the landowner to minimize the danger of incurring significant financial losses if the practice proves to be unprofitable or fails due to inexperience. The reductions in uncertainty and risk from trialability contribute to enhanced adoption for most people who are psychologically averse to risk and uncertainty (e.g., ref. [66]).

Furthermore, trialability includes the divisibility of an innovation (its use on a small scale),^[^ ^12^, ^67^ ^]^ observability of results from innovation,^[^ ^12^, ^68^ ^]^ where the higher the observability and a shorter lag for observability the fewer the trials necessary to sufficiently reduce uncertainty to make a choice between adoption and nonadoption; complexity where the greater the complexity, the greater the information that landholders require to be certain about the consequences of adopting it;^[^ ^12^ ^]^ the cost of undertaking a trial; and threats to a biological trial, e.g., failure. The following section has a compilation and discussion of some of the characteristics of fertilizer that may influence its rate of adoption by farmers concerning its relative advantage and trialability.

## 13 Price of Fertilizer

The price or cost of fertilizer is one of the reasons why farmers are not using fertilizer.^[^ ^2^, ^22^, ^29^, ^48^ ^]^ Nunoo et al.^[^ ^29^ ^]^ reported that 46.5% of the farmer respondents claimed they could not afford fertilizer because the cost of fertilizer is too high, while Okoboi & Barungi^[^ ^2^ ^]^ noted that the most frequently cited reason, at 50% of respondents, for not using inorganic fertilizers was that they are too expensive. Furthermore, the price of fertilizer and farm size is significant; thus, high and large farm sizes are disincentive to adoption.^[^ ^29^ ^]^ Farm size and fertilizer price are critical in determining the quantity of fertilizer used. Morris et al.^[^ ^52^ ^]^ and Jaja and Barber^[^ ^69^ ^]^ observe that the demand for fertilizer is frequently low in Africa because of the low fluctuating crop yields, on the one hand, and the high fertilizer prices relative to crop prices on the other, both of which diminish incentives to use fertilizer. This is consistent with,^[^ ^48^ ^]^ who found that high fertilizer prices with low crop/output prices lessen farmers' demand for fertilizers. The high farm‐level fertilizer prices cannot be offset by produce prices even with a significant yield response to fertilizer application. Similarly, the price of fertilizer compared to the price of the produce creates adoption challenges, especially for small‐scale farmers who are not gaining from margins of scale and will not be profitable.^[^ ^2^ ^]^ These elements undermine the fertilizer's reliability, compatibility, and relative advantage, impacting how widely it is used.

## 14 Labor Requirements

Innovation adoption is positively related to labor resources^[^ ^13^ ^]^: the labor requirement in fertilizer use makes it less attractive to farmers constrained in resources like income, hired labor, and mechanization. Green and Ng'ong'ola^[^ ^50^ ^]^ listed regular labor availability as the primary influence on Malawians' adherence to fertilizer recommendations. Both availability and cost of labor are key in adopting innovation. They would result in farmers' low adoption of the technology due to a lack of resources, such as money and labor, to apply them. These aspects further present fertilizer as having a lower relative advantage and high complexity, reducing its adoption.

## 15 The Bulkiness

The way fertilizer is packed and how much volume it occupies, coupled with the distance to farm plots or from fertilizer supplier shops, in addition to farm size, road conditions, means of transport, age, and gender, might work as a disincentive for adoption by certain categories of farmers.^[^ ^43^, ^48^, ^70^, ^71^ ^]^ This is because farmers who have farms distant from their communities—or face physical difficulties resulting from old age or other limitations—frequently have difficulty acquiring and transporting inputs to their farms, which is likely to have a detrimental impact on the adoption of a bulky or heavy agricultural technology.^[^ ^71^ ^]^ The road conditions might also not support the efficient movement of overly loaded vehicles.^[^ ^48^ ^]^ Together, these elements undermine the fertilizer's reliability, trialability, and compatibility while increasing its complexity, impacting how widely it is used.

## 16 Availability of Fertilizer

Nunoo et al.^[^ ^29^ ^]^ reported that the reason farmers gave for not using fertilizer was its unavailability, and poor access.^[^ ^22^ ^]^ This is no exception to inorganic fertilizers, given that most developing countries import these fertilizers and quickly run out of stock—if available on the market. Ref. [2] note that 14.1% of the respondents did not use inorganic fertilizer because they lacked access to fertilizer. This unavailability of fertilizer decreases its use and the chance for adoption, compromising the trialability and reliability of fertilizer as an innovation.

## 17 Requires Technical Know‐How

Farmers' reasons for their low adoption of the technologies also involve technical know‐how requirements.^[^ ^72^ ^]^ Fertilizer use requires accuracy and precision for nutrient management. This involves applying the right source of fertilizer at the correct rate, at the right time, and in the right place.^[^ ^73^ ^]^ Anang^[^ ^4^ ^]^ reports that before applying fertilizer, the aspects mentioned above are important for its efficiency. If fertilizer is used wrongly, it can harm the crop or result in lower yields and losses. These requirements can be difficult for potential adopters to follow and successfully implement on the first try. There is also inadequate technical information provided to small‐holders on how they might make profitable use of inorganic fertilizer or explain why fertilizer fails, so farmers quickly turn away from using the technology. This lack of crop‐specific agronomic and economic information on fertilizers impacts potential adopters' perceptions. It compromises the reliability of fertilizer, its relative advantage, observability, trialability, and compatibility while increasing complexity as an innovation.^[^ ^12^ ^]^ Suppose the technology or practice used in the trial is implemented poorly; in that case, the trial will be less likely to meet this requirement of observability and learning even after time and financial investments. Acquainting farmers to fertilizer and its proper use is worthwhile^[^ ^22^ ^]^ for increased adoption.

## 18 Conclusion 

Numerous studies on the variables influencing adoption decisions vary widely concerning one factor's importance over another. Furthermore, there are regional differences in the sociocultural and economic contexts that farmers operate in, in addition to the heterogeneity of adopters and their circumstances, which increases the possibility that the findings of those studies may differ. Understanding producers' socio‐economic characteristics and perceptions of innovation is therefore vital to understanding and increasing the adoption of agricultural technologies and best management practices and has been widely studied. However, it is essential to also consider the characteristics of the innovation itself—its relative advantage and trialability, among other characteristics, have also been found to impact adoption rates, as this seems to be the unique aspect of why many innovations are not being adopted.

Fittingly, vast and sometimes obfuscated documentation of adopters' characteristics and innovations may form a basis for gaining insights and designing beneficial interventions for improving adoption rates. This literature review attempts to identify and describe those that have been described as relevant to fertilizer adoption in sub‐Saharan Africa. We found evidence of socio‐economic characteristics influencing adoption, including farmers' perceptions of fertilizer, access to information, farm size, farmer age (experience in cocoa growing), farm income, access to credit, and farmer gender. We additionally identified several characteristics of fertilizer that influence its adoption: price, labor requirements, bulkiness and distance to major markets, availability, and technical knowledge required. While all of these factors were found to have various impacts on adoption, farm income, access to credit, labor requirements, and fertilizer price/availability stood out as particularly influential. Specifically, higher farm income and access to credit led to higher adoption rates, while high prices, low availability, and high labor requirements were considered significant barriers. One gap in this literature was the lack of categorization of the respondents into the stages of adoption based on their positions on a spectrum from innovators to laggards; farmers were often considered as binary adopters or nonadopters, with minimal regard for the pace of adoption and what impacted this.

To clearly understand the reasons for the low rates of fertilizer adoption in sub‐Sarahan African cocoa farming, it is necessary to consider a wide range of factors—including farmer characteristics and characteristics of the innovation—as they each intertwine with and influence each other. For example, farmers' gender influenced adoption in various ways, from playing a role in farm size and resource access to interacting with the bulkiness of fertilizer to impact relative advantage and trialability. We therefore encourage future studies, including extension and outreach efforts, to carefully consider the interplay of a multitude of personal, agronomic, and technological factors when exploring and promoting fertilizer adoption in cocoa farming. We also suggest that future research explore categorizing respondents into the stages of adoption based on their positions on a spectrum from innovators to laggards for each factor documented, as this would provide more insights into temporal adoption patterns.

## 19 Recommendation to Increase Fertilizer Adoption by Cacao Farmers

Research and extension need to develop fertilizer recommendations specific to cacao production for the different soils.

Strengthen government programs for community‐led research and increased exposure opportunities for target audiences. For example, having government agencies establish demonstration sites and learning centers where farmers can freely observe and share experiences with peers and researchers with expertise.

Continuous research and extension will help demystify negative beliefs and attitudes and change perceptions toward innovations, thereby increasing knowledge through training and education programs about proposed innovations.^[^ ^22^ ^]^

There is a need to define and identify target audiences and align innovations with farmers' characteristics like abilities and capabilities to adopt, production seasons, cultural values and beliefs, socio‐economic diversities, and enabling environment to ease interaction of farmers with services (marketing, community support, transport, and processing) and inputs.^[^ ^26^ ^]^

Aligning and having a target audience for a given innovation will help in realistic evaluations of adoption and may result in reporting it differently. For instance, adoption could be measured for a specific audience in a specified period.

There is a need for designing and implementing policies that are more inclusive of all farmer's priorities and practices, economic and social levels, for increased fertilizer accessibility, affordability, and profitability.^[^ ^22^ ^]^

There is a need to increase awareness among farmers of cacao's economic value, susceptibility of soil to nutrient depletion and degradation, indicators of soil fertility, and ways for soil fertility enhancement. This will create opportunities for farmers to find ways of boosting the yield, hence increasing fertilizer use.

## Conflict of Interest

The authors declare no conflict of interest.
